# Mitochondrial Genome of *Strophopteryx fasciata* (Plecoptera: Taeniopterygidae), with a Phylogenetic Analysis of Nemouroidea

**DOI:** 10.3390/genes13071116

**Published:** 2022-06-22

**Authors:** Xuan Guo, Caiyue Guo, Xiaojiao Dong, Heng Zhang, Dávid Murányi, Weihai Li, Ying Wang

**Affiliations:** 1 Henan International Joint Laboratory of Taxonomy and Systematic Evolution of Insecta, Henan Institute of Science and Technology, Xinxiang 453003, China; guoxuan1999@163.com (X.G.); gcy159365@163.com (C.G.); r1se060812r1se@163.com (X.D.); zyh31355555@163.com (H.Z.); lwh7969@163.com (W.L.); 2 Plant Protection Institute, Centre for Agricultural Research, Hungarian Academy of Sciences, Herman Ottó út 15, H-1022 Budapest, Hungary; d.muranyi@gmail.com; 3 Department of Zoology, Hungarian Natural History Museum, Baross u. 13, H-1088 Budapest, Hungary

**Keywords:** mitochondrial genome, Taeniopterygidae, phylogeny

## Abstract

Taeniopterygidae is a medium-sized family of stoneflies. The phylogeny of Taeniopterygidae was widely accepted based on the morphological analyses. However, there are different opinions based on molecular data. To date, only two taeniopterygid mitochondrial genomes (mitogenomes) were available, and more sampling is needed to obtain precise phylogenetic relationships. In this research, the *Strophopteryx fasciata* mitogenome was sequenced and analyzed. The complete mitogenome of *S. fasciata* was 15,527 bp in length and contained 37 genes and a non-coding control region. Among taeniopterygid mitogenomes, the length variation was minimal in protein-coding genes (PCGs), transfer RNA genes (tRNAs) and ribosomal RNA genes (rRNAs), but very different in the control region. Similar to mitogenomes of other taeniopterygid species, the *S. fasciata* mitogenome was consistently AT biased and displayed positive AT- and negative GC-skews of the whole mitogenome. Most PCGs used ATN as the start codon and TAA/TAG as the stop codon. The stop codons were far less variable than the start codons in taeniopterygid mitogenomes. All *Ka*/*Ks* ratios were less than 1, indicating the presence of purifying selection in these genes. The secondary structures of transfer and ribosomal RNA genes of *S. fasciata* mitogenome are highly conserved with other taeniopterygid species. In the control region of the *S. fasciata* mitogenome, some essential elements (tandem repeats, stem–loop structures, and poly−N stretch, etc.) were observed. Two phylogenetic trees were inferred from Bayesian inference (BI) and Maximum Likelihood (ML) methods generated the identical topology across the PCGR dataset. The relationships of five families in Nemouroidea were recovered as Leuctridae + ((Capniidae + Taeniopterygidae) + (Nemouridae + Notonemouridae)). These results will help us understand the mitogenome structure of taeniopterygid species and the evolutionary relationship within Plecoptera.

## 1. Introduction

Taeniopterygidae is a medium-sized family of stoneflies (Plecoptera) consisting of about 75 species with Holarctic distribution [1,2]. They are found in all sizes of streams but are probably most species rich in larger ones [1]. Currently, the most widely accepted classification system of stonefly was proposed by Zwick (2000) based on the morphological analyses [2]. In Zwick’s (2000) study, Taeniopterygidae and other four families (Capniidae, Leuctridae, Nemouridae and Notonemouridae) were assigned to the superfamily Nemouroidea [2]. The family Taeniopterygidae can be easily distinguished with other Nemouroidea families by the elongated second tarsal segment [3]. At the family level, the Taeniopterygidae is recognized as monophyletic, and is placed as the most basal lineage of Nemouroidea [2]. However, there are different opinions based on molecular data. Based on 18S sequence data, Thomas et al. supported Nemouridae as the earliest branch within Plecoptera [4]. Terry used six molecular markers to construct the phylogeny of Plecoptera [5]. The results supported the basal position of Leuctridae and the sister-group relationship of Taeniopterygidae and Scopuridae [5]. In addition, based on transcriptome data, Davis listed Taeniopterygidae and Capniidae as sister families [6]. This result was also supported by most mitochondrial genomic studies [7,8,9,10,11,12]. Recently, South et al. recovered the phylogeny of the North American Plecoptera using sequences of single-copy orthologous genes selected from transcriptomes [13]. However, inconsistent relationships of Taeniopterygidae were generated from the analysis of different datasets [13].

Currently, mitochondrial genomes (mitogenomes) are commonly employed for phylogenetic analysis of insect lineages because of their maternal inheritance, rare recombination, relatively high evolutionary rate, and conserved gene components [14,15,16]. With the development of next-generation sequencing technologies, large numbers of mitogenomes have been extensively employed to resolve deep-level phylogenetic relationships [17,18,19].

To date, there are 86 complete or near complete plecopteran mitogenomes available in GenBank (as of May 2022). Among these sequences, however, only two complete mitogenomes representing the Taeniopterygidae were available. Relevant phylogenetic studies have been carried out to investigate the molecular phylogenetic relationships among stoneflies. However, taxon sampling in these studies has been limited and is not enough to obtain precise phylogenetic relationships within Nemouroidea.

In this study, the complete mitogenome of *Strophopteryx fasciata* was sequenced. The mitochondrial genomic structure and composition, such as gene content and RNA secondary structure, were also compared with other taeniopterygid mitogenomes. Furthermore, the phylogeny of Nemouroidea was reconstructed using published mitogenomes along with this newly sequenced mitogenome. The results will help us understand the evolutionary relationship within Plecoptera.

## 2. Materials and Methods

### 2.1. Sample Collection, DNA Extraction and Sequencing

Specimens of *S. fasciata* were collected from Putnam County in Georgia, USA. Specimens were preserved in 100% ethanol and stored at −20 °C. Total genomic DNA was extracted from adults using the DNeasy tissue kit (Qiagen, Hilden, Germany). The voucher specimens are kept in Henan International Joint Laboratory of Taxonomy and Systematic Evolution of Insecta, Henan Institute of Science and Technology (HIST), Xinxiang, China. The DNA concentration was measured for each sample by using NanoDrop One (Thermo Scientific, Waltham, MA, USA). DNA samples with qualified concentration (>10 ug) were sent to Berry Genomics Co., Ltd. (Beijing, China) for further detecting. 

An Illumina TruSeq library was prepared with an insert size of 480 bp and was sequenced on the Illumina Hiseq 2500 platform with 250 bp paired-end reads. Raw reads were checked by FastQC 0.11.3 [20], with adapters and low-quality reads filtered by Trimmomatic [21]. A total of 6 GB clean data were obtained and used in the de novo assembly using IDBA-UD [22] with minimum and maximum k values of 45 bp and 145 bp. To identify the mitogenome sequences, the contigs obtained were searched with the *COI* and *srRNA* gene sequences using BLAST with at least 98% similarity. The complete mitogenome assembling strategy is similar to that of our previous studies [8,9,23,24,25].

### 2.2. Genome Annotation and Sequence Analysis

Geneious 6.1.6 was used to assemble Illumina sequence reads into contigs [26]. Then, the assembled mitochondrial sequences were annotated with MITOS [27]. All transfer RNA genes (tRNAs) were identified by MITOS. The secondary structure of the tRNA gene was also predicted by MITOS. Protein-coding genes (PCGs) and two ribosomal RNA (rRNA) genes were verified by alignment with homologous genes from other published stonefly species. The graphical mitogenomic map was depicted with OGDRAW v1.3.1 [28]. The MAFFT algorithm (within TranslatorX online) was used for the alignment of each PCG, using codon–based multiple alignment [29]. Before the protein alignment was back translated to nucleotides, GBlocks (in TranslatorX) with default settings were used to remove ambiguously aligned areas. The G–INS–I alignment strategy in MAFFT 7.0 online was used for rRNA alignment [30], and ambiguously aligned regions masked with Gblocks [31].

Nucleotide composition and codon usage were calculated by MEGA 6.0 [32]. AT and GC asymmetries were calculated using the following formulas: AT skew = [A − T]/[A + T] and GC skew = [G − C]/[G + C] [33]. DnaSP [34] was used to calculate the value of Ka (the nonsynonymous substitution rate) and Ks (the synonymous substitution rate). Tandem Repeats Finder server [35] and mfold web server [36] were used to identify tandem repeats and to infer the stem−loop structure, respectively.

### 2.3. Phylogenetic Analysis

To investigate the phylogenetic relationships within the Nemouroidea, 27 Nemouroidea species with published mitogenomes and the newly sequenced mitogenome were used in this study. Outgroups used two perlodid species, *Isoperla eximia* and *Pseudomegarcys japonicus* (Table 1). The PCGR dataset (13,194 bp, including all 13 PCGs plus two rRNA genes) was assembled for phylogenetic analyses.

GTR+I+G was selected as the best−fit nucleotide substitution model for each gene using ModelFinder applying the Akaike Information Criterion (AIC) [37]. Maximum Likelihood (ML) phylogenetic trees were inferred using IQ–TREE [37], and an ultrafast bootstrap approximation with 1000 replicates. Bayesian analyses were carried out using MrBayes v3.2.6 with selected models [38]. MrBayes runs were as follows: 10 million generations with four chains, sampling every 100 generations, and the first 25% discarded as burn–in.

## 3. Results and Discussion

### 3.1. General Features of Mitogenome

**Figure 1:**
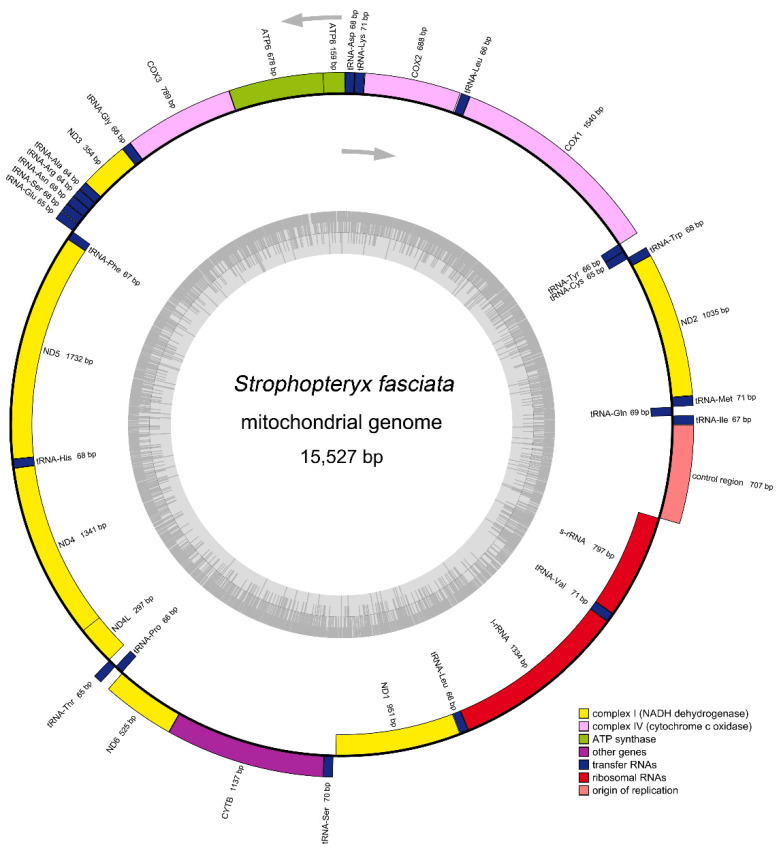
Circular map of the *S. fasciata* mitogenome. Genes shown on the inside of the map are transcribed in a clockwise direction, whereas those on the outside of the map are transcribed counterclockwise.

The complete mitogenome of *S. fasciata* (15,527 bp) was determined (GenBank accession ON500674; Figure 1). The *S. fasciata* mitogenome was a traditional circular DNA molecule and medium-sized when compared with mitogenomes of other taeniopterygid species, which typically ranged from 15,353 bp to 16,020 bp (Table 1). This mitogenome was the second smallest one among the Nemouroidea complete mitogenomes (Table 1). Among taeniopterygid mitogenomes, the length variation was minimal in PCGs, tRNAs and rRNAs, but very different in the control region (Table 2). The mitogenome of *S. fasciata* contained 37 genes (22 tRNAs, 13 PCGs and 2 rRNAs) and a control region (Figure 1 and Table S1), which were typically present in metazoan mitogenomes [39]. The gene order of the *S. fasciata* mitogenome was identical with the ancestral gene order of *Drosophila yakuba*, which was thought to be the ground pattern for insect mitogenomes [40]. All three taeniopterygid mitogenomes had highly conserved gene order, other Nemouroidea mitogenomes having the insect ancestral gene order [7,8,9].

A total of 45 bp overlapping nucleotides were found at 14 gene junctions; the longest overlap (8 bp) existed between *tRNA^Trp^* and *tRNA^Cys^*, and *tRNA^Tyr^* and *COI* (Table S1). Except for the large non-coding control region, the *S. fasciata* mitogenome also included seven intergenic spacers, ranging in size from 1 to 16 bp (Table S1).

### 3.2. Nucleotide Composition and Codon Usage

Similar to other taeniopterygid mitogenomes, *S. fasciata* mitogenome was consistently AT biased, with an A + T value of 68.1%, and displayed positive AT- (0.03) and negative GC-skews (−0.18) of the whole mitogenome (Table 2). In all taeniopterygid species, both PCGs and rRNAs had a negative AT-skew, and the control region had a positive AT-skew. Meanwhile, the rRNAs had a positive GC-skew, and the control region had a negative GC-skew (Table 2). In addition, the control region showed a large variation in AT- (from 0.01 to 0.07) and GC-skews (from −0.16 to −0.30) (Table 2).

**Figure 2:**
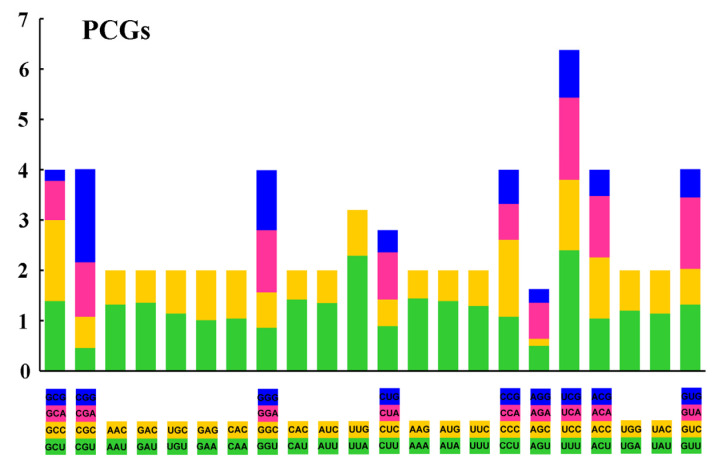
The relative synonymous codon usage (RSCU) in the mitogenome of *S. fasciata*. PCGs represent protein-coding genes.

The codon usage pattern in all investigated taeniopterygid mitogenomes is consistent with the typical invertebrate mitochondrial genetic code, which favors AT-rich codons. In this study, the value of relative synonymous codon usage (RSCU) was calculated. In the PCGs of *S. fasciata* mitogenome, A or T were overwhelmingly represented compared to G or C at the third codon position (Figure 2 and Table S2). Four most frequently used codons in *S. fasciata* were TTA, ATT, TTT, and ATA, and they were all composed wholly of A and/or T (Table S2). Overall, the nucleotide compositions and codon usage of *S. fasciata* were almost the same as the other two taeniopterygid mitogenomes.

### 3.3. Protein-Coding Genes

The full length of *S. fasciata* PCGs was 11,193 bp (excluded the stop codon), with an A + T content of 66.9% (Table 2). In *S. fasciata* mitogenome, twelve PCGs used the typical start codon ATN (three started with ATT, and nine started with ATG). Meanwhile, *ND1* gene used TTG as the start codon (Table 3 and Table S1). Ten PCGs used the standard stop codon TAA or TAG (seven stopped with TAA, and three stopped with TAG), while the remaining three PCGs (*COI*, *COII*, and *ND5*) were terminated with an incomplete stop codon T (Table 3 and Table S1).

In taeniopterygid mitogenomes, eight PCGs initiated with the same start codon, but five PCGs used multiple start codons (both *ND2* and *ND5* with ATG and GTG, *ATP8* with ATT and ATC, *ND6* with ATG and ATT, and *CytB* with ATG and ATC) (Table 3). The unusual start codons TTG and GTG also existed in other Nemouroidea mitogenomes [7,8,9,10,11]. The stop codons were far less variable than the start codons in taeniopterygid mitogenomes. Most PCGs used the same stop codons, except for the *CytB* gene, which used TAA in *Taeniopteryx ugola* and TAG in other two taeniopterygid species (Table 3). In addition, we found that three PCGs (*COI*, *COII* and *ND5*) in all three taeniopterygid mitogenomes used truncated stop codons (Table 3). Insect mitogenomes frequently have incomplete stop codons, which are thought to be repaired by polyadenylation after transcription [41,42].

**Figure 3:**
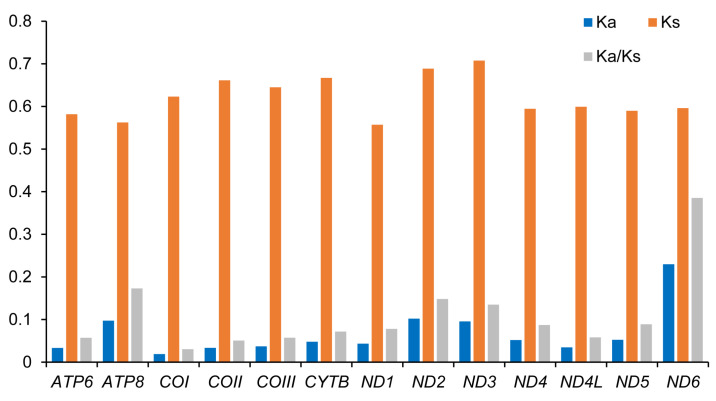
Evolutionary rates of taeniopterygid mitogenomes. The non-synonymous substitutions rate (Ka), the synonymous substitutions rate (Ks), and the ratio of the rate of non-synonymous substitutions to the rate of synonymous substitutions (Ka/Ks) for each PCG.

The nonsynonymous substitutions rate (Ka), the synonymous substitutions rate (Ks), and the ratio of Ka/Ks (ω) for each PCG in three taeniopterygid species were calculated (Figure 3). In all PCGs, *ND3* showed the highest Ks, whereas *ND6* showed the highest Ka and ω values (Figure 3). The ratios of Ka/Ks were all less than 1, indicating the existence of purifying selection in these genes [43]. Therefore, all mitochondrial PCGs could be used to analyze the phylogeny of these species in Taeniopterygidae.

### 3.4. Transfer and Ribosomal RNA Genes

**Figure 4:**
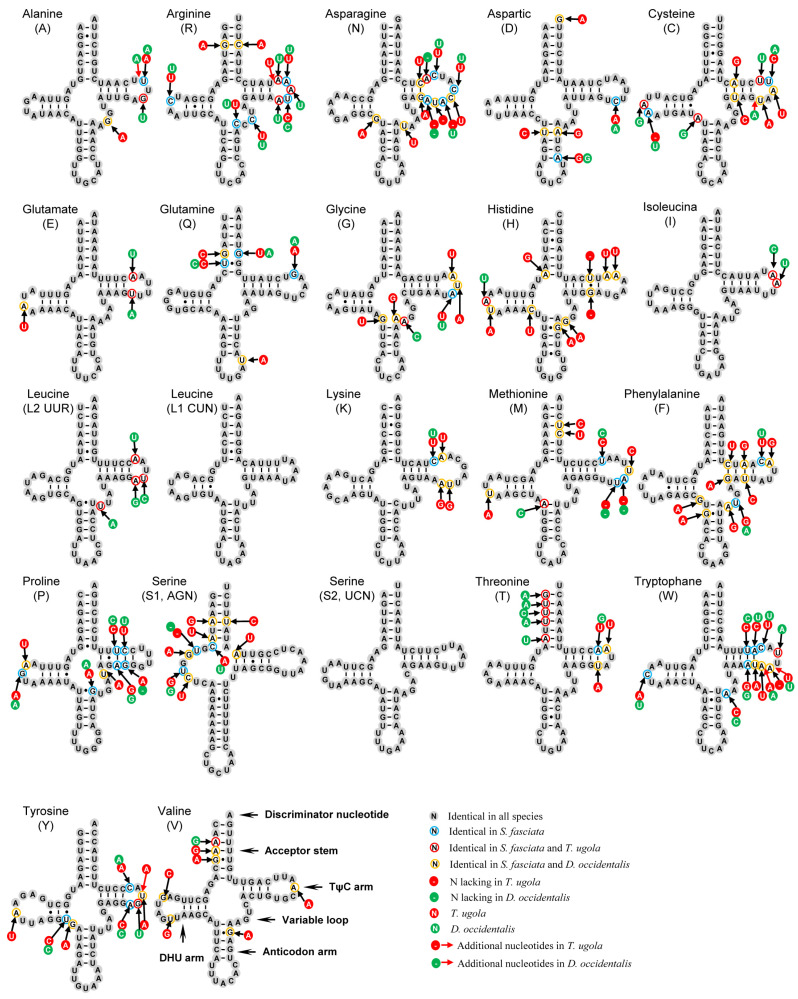
Predicted secondary structures of 22 tRNAs in *S. fasciata*. Conserved sites within three taeniopterygid species are indicated as black nucleotides within gray spheres. Red arrows correspond to insertions.

The 22 tRNAs in *S. fasciata* mitogenome were of a total length of 1479 bp, ranging in size from 64 to 71 bp (Table 2 and Table S1). The typical clover-leaf structure was found in 21 tRNAs, except for *tRNA^Ser(AGN)^* whose dihydrouridine (DHU) arm is absent (Figure 4). This phenomenon is a typical feature of metazoan mitogenomes [44]. A total of 32 mismatched base pairs were detected in *S. fasciata* tRNAs based on the secondary structures, such as 26 bp weak G–U pairs, 1 bp U–C mismatch, 1 bp A–G mismatch, 1 bp G–G mismatch and 3 bp A–C mismatches (Figure 4). By contrast, the secondary structures showed the most conserved tRNAs in taeniopterygid mitogenomes were *tRNA^Leu(CUN)^*, *tRNA^Ser(UCN)^*, *tRNA^Ile^*, *tRNA^Ala^* and *tRNA^Glu^* (less than three nucleotides substitution per gene). In addition, the most conserved and variable regions were found in the anticodon arm and TΨC loop, respectively (Figure 4).

The large subunit ribosome gene (*lrRNA*) and small subunit ribosome gene (*srRNA*), which were typically found in other insect mitogenomes, were also observed in *S. fasciata* mitogenome sequenced in this study. The size of *lrRNA* and *srRNA* of *S. fasciata* was 1334 bp and 797 bp, respectively (Table S1). The length of rRNA genes varied from 2119 bp in *T. ugola* and 2131 bp in *S. fasciata*. The A + T content ranged from 71.2% in *S. fasciata* to 72.7% in *T. ugola* (Table 2). The *lrRNA* gene had six domains (domain III was absent in arthropods) and 44 helices (Figure S1). Nucleotide variability was unevenly distributed among domains and helices, mainly in domains I, II and VI. Several helices (H837, H845, H991, and H2077) in *lrRNA* showed high variability at the primary sequence level (Figure S1). The *srRNA* gene had three domains and 27 helices (Figure S2). Nucleotide variability was unevenly distributed among domains and helices, mainly in helices H47 of domain I (Figure S2). 

Overall, the secondary structures of tRNA and rRNA genes of *S. fasciata* mitogenome are highly conserved. These conserved regions may be involved in the structure and function of RNAs [8,45].

### 3.5. The Control Region

**Figure 5:**
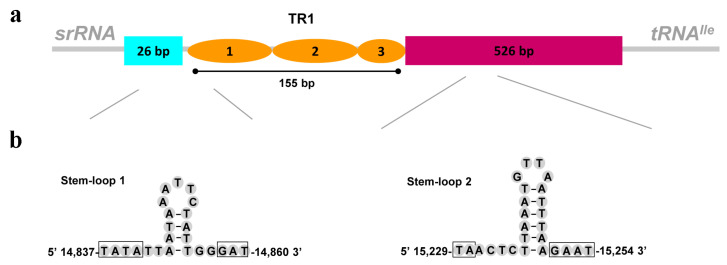
Organization of the control region in *S. fasciata* mitogenome. (**a**) control region structure of *S. fasciata.* TR is the abbreviation of tandem repeat units; (**b**) putative stem–loop structures found in the control region of *S. fasciata*.

The length of the control region was highly variable in taeniopterygid mitogenomes, ranging from 537 bp in *T. ugola* to 1248 bp in *D. occidentalis*. The A + T content ranged from 71.0% in *S. fasciata* to 78.9% in *T. ugola* (Table 2). In the control region of *S. fasciata* mitogenome, some essential elements were observed: (1) a 26 bp region that was bordered by *srRNA*; (2) a large tandem repeat sequence (155 bp); and (3) a 526 bp region at the end of the control region (Figure 5a). The tandem repeat sequence included two tandem repeat units plus a partial copy of the repeat. One microsatellite sequence ((TA)5, position 15,184 bp–15,193 bp) was also found in the control region. Microsatellite elements can be used to examine the geographical structure and phylogenetic relationship of species [45].

The stem-loop (SL) structure in *Drosophila* control region is considered to be the initiation site for secondary chain synthesis [46]. Among some insects, the SL structure showed highly conserved flanking sequences, such as ‘TATA’ sequence at the 5’ end and ‘G(A)nT’ at the 3’ end [47,48]. In this study, two SL structures were found in the control region (Figure 5b). Both flanking ‘TATA’ sequence at the 5’ end and ‘GAT’ at the 3’ end were found in the SL1 (position 14,837 bp–14,860 bp). However, in the SL2 (position 15,229 bp–15,254 bp), only conserved flanking ‘GAAT’ was found at the 3’ end (Figure 5b).

Several poly-N stretch (≥7 bp), such as poly-T (position 15,107 bp–15,027 bp), poly-A (position 15,421 bp–15,427 bp) and poly-C (position 15,483 bp–15,489 bp), were found in the control region of *S. fasciata*. The poly-T stretch is relatively conserved across insects [49], and it could play a role in transcriptional regulation or serve as a replication start site. [46]. Most of these structure elements are also identified in other taeniopterygid species [7] and stoneflies [8,9,10,11,23,24,25,50,51,52].

### 3.6. Phylogenetic Analysis

**Figure 6:**
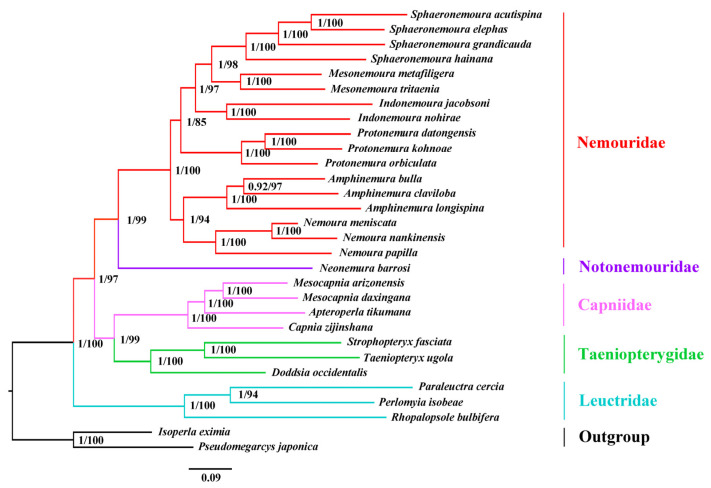
Phylogenetic tree inferred from the sequences of 13 PCGs plus two rRNAs of the mitochondrial genomes of 28 Nemouroidea species and two outgroups. Numbers at nodes are Bayesian posterior probabilities (**left**) and ML bootstrap values (**right**).

Two phylogenetic trees were inferred from BI and ML methods and generated the identical topology across the PCGR dataset (Figure 6). The monophyly of each family was well-supported (Bootstrap values (BP) > 97, post-probability values (PP) = 1.00), except for Notonemouridae, which only possesses one species. *Strophopteryx fasciata* and *T. ugola* were together (BP = 100, PP = 1.00) and were sister to *D. occidentalis* (BP = 100, PP = 1.00). 

In Nemouridae, the subfamily Amphinemurinae was recovered as paraphyletic. The genus *Amphinemura* that belongs to Amphinemurinae was sister to *Nemoura* of the subfamily Nemourinae (PP = 94, BP = 1.00). The sister relationship between *Amphinemura* and *Nemoura* is not consistent with the traditional classification [53] but consistent with some mitogenome analyses [8,54]. Considering that only one genera data of Nemourinae is available, more sampling from different genera of this subfamily is needed to test this problem.

This analysis supported Nemouridae as the sister taxon to Notonemouridae (BP = 99, PP = 1.00) (Figure 6). This placement disagrees with some molecular analyses [4,5] but is consistent with previous morphological hypothesis [2] and mitogenome analyses [8,9,11,55]. In Zwick’s study, Capniidae was placed as a sister to Leuctridae and then clustered with Nemouridae plus Notonemouridae [2]. However, this analysis supported a sister relationship between Capniidae and Taeniopterygidae (BP = 99, PP = 1.00) (Figure 6). Although this result differs from the generally accepted hypothesis that the Capniidae and Leuctridae are sister groups [2], there are no molecular studies supporting this hypothesis until now. 

Like previous mitogenome studies [8,9], the clade Capniidae + Taeniopterygidae and the clade Chloroperlidae + Perlodidae were grouped together. However, results in this study showed higher stability (BP = 97, PP = 1.00) than previous mitogenome studies (BP = 47/34, PP = 0.56/0.95) [9,12]. In addition, the Leuctridae was the earliest branch within the superfamily Nemouroidea in this study (BP = 100, PP = 1.00). Finally, the relationships of five families in Nemouroidea were recovered as Leuctridae + ((Capniidae + Taeniopterygidae) + (Nemouridae + Notonemouridae)).

## 4. Conclusions

Nowadays, the phylogeny of Taeniopterygidae was widely accepted based on morphological analyses. However, there are different opinions based on molecular data. Considering only two taeniopterygid mitogenomes are available, more sampling is needed to obtain precise phylogenetic relationships. In this study, the complete mitogenome of *S. fasciata* was sequenced and analyzed. The complete mitogenome of *S. fasciata* was a traditional circular DNA molecule and medium-sized when compared with mitogenomes of other taeniopterygid species. Compared with other taeniopterygid mitogenomes, the *S. fasciata* mitogenome showed itself to be highly conserved in mitogenome size, gene order, nucleotide composition, codon usage and RNA secondary structures. In the control region of the *S. fasciata* mitogenome, some essential elements (tandem repeats, stem–loop structures, and poly-N stretch, etc.) were observed. Two phylogenetic trees were inferred from BI and ML methods and generated the identical topology across the PCGR dataset. *Strophopteryx fasciata* and *T. ugola* were together and were sister to *D. occidentalis*. The relationships of five families in Nemouroidea were recovered as Leuctridae + ((Capniidae + Taeniopterygidae) + (Nemouridae + Notonemouridae)). These results will help us understand the mitogenome structure of taeniopterygid species and the evolutionary relationship within Plecoptera.

## Figures and Tables

**Table 1:** General information of nemourid species used in this study.

Family	Species	Number (bp)	Accession Number
Capniidae	*Apteroperla tikumana*	15,564	NC_027698
	*Capnia zijinshana*	16,310	KX094942
	*Mesocapnia arizonensis*	14,921	KP642637 *
	*Mesocapnia daxingana*	15,524	KY568983 *
Leuctridae	*Rhopalopsole bulbifera*	15,599	MK111419 *
	*Paraleuctra cercia*	15,625	MK492251
	*Perlomyia isobeae*	15,795	MK492252
Nemouridae	*Nemoura meniscata*	15,895	MN944386
	*Nemoura nankinensis*	16,602	KY940360
	*Nemoura papilla*	15,774	MK290826
	*Amphinemura longispina*	15,709	MH085446
	*Amphinemura bulla*	15,827	MW339348
	*Amphinemura claviloba*	15,707	MN720741
	*Indonemoura jacobsoni*	15,642	MH085448
	*Indonemoura nohirae*	15,738	MH085449
	*Mesonemoura metafiligera*	15,739	MH085450
	*Mesonemoura tritaenia*	15,778	MH085451
	*Protonemura kohnoae*	15,707	MH085452
	*Protonemura orbiculata*	15,758	MH085453
	*Protonemura datongensis*	15,756	MT276842
	*Sphaeronemoura elephas*	15,846	MN944385
	*Sphaeronemoura grandicauda*	15,661	MH085454
	*Sphaeronemoura acutispina*	15,016	MH085455 *
	*Sphaeronemoura hainana*	15,260	MK111420 *
Notonemouridae	*Neonemura barrosi*	14,852	MK111418 *
Taeniopterygidae	*Taeniopteryx ugola*	15,353	MG589786
	*Doddsia occidentalis*	16,020	MG589787
	*Strophopteryx fasciata*	15,527	ON500674
Perlodidae (Outgroup)	*Isoperla eximia*	16,034	MG910457
	*Pseudomegarcys japonica*	16,067	MG910458

* Incomplete mitogenome sequence.

**Table 2:** Mitochondrial nucleotide composition in three taeniopterygid species.

Region	Feature	*S. fasciata*	*D. occidentalis*	*T. ugola*
Whole mitgenome	Size (bp)	15,527	16,020	15,353
	A + T%	68.1	68.4	69.8
	AT-skew	0.03	0.02	0.02
	GC-skew	−0.18	−0.21	−0.15
Protein-coding genes	Size (bp)	11,193	11,148	11,223
	A + T%	66.9	66.8	68.6
	AT-skew	−0.19	−0.20	−0.20
	GC-skew	−0.03	−0.01	0.03
tRNAs	Size (bp)	1479	1477	1471
	A + T%	70.9	70.8	71.3
	AT-skew	0.03	−0.01	−0.02
	GC-skew	−0.13	0.13	0.13
rRNAs	Size (bp)	2131	2126	2119
	A + T%	71.2	72.4	72.7
	AT-skew	−0.05	−0.06	−0.05
	GC–skew	0.28	0.28	0.28
Control region	Size (bp)	707	1248	537
	A + T%	71.0	72.7	78.0
	AT-skew	0.07	0.01	0.02
	GC-skew	−0.16	−0.30	−0.20

**Table 3:** Start and stop codons of three taeniopterygid mitogenomes.

Gene	Start Codon			Stop Codon		
	*S. fasciata*	*T. ugola*	*D. occidentalis*	*S. fasciata*	*T. ugola*	*D. occidentalis*
*ND2*	ATG	GTG	ATG	TAA	TAA	TAA
*COI*	ATT	ATT	ATT	T-	T-	T-
*COII*	ATG	ATG	ATG	T-	T-	T-
*ATP8*	ATT	ATC	ATT	TAA	TAA	TAA
*ATP6*	ATG	ATG	ATG	TAA	TAA	TAA
*COIII*	ATG	ATG	ATG	TAA	TAA	TAA
*ND3*	ATT	ATT	ATT	TAG	TAG	TAG
*ND5*	ATG	GTG	ATG	T-	T-	T-
*ND4*	ATG	ATG	ATG	TAA	TAA	TAA
*ND4L*	ATG	ATG	ATG	TAA	TAA	TAA
*ND6*	ATG	ATT	ATT	TAA	TAA	TAA
*CYTB*	ATG	ATG	ATC	TAG	TAA	TAG
*ND1*	TTG	TTG	TTG	TAG	TAG	TAG

## Data Availability

The data that support the findings of this study are deposited in GenBank with accession number ON500674. The data are available from the corresponding authors upon reasonable request.

## Supplementary Materials

The following supporting information can be downloaded at: https://www.mdpi.com/article/10.3390/genes13071116/s1, Figure S1: Predicted secondary structure of the *lrRNA* gene in the *S. fasciata* mitogenome; Figure S2: Predicted secondary structure of the *srRNA* gene in the *S. fasciata* mitogenome; Table S1: Organization of the *S. fasciata* mitochondrial genome; Table S2: Codon number and RSCU in the *S. fasciata* mitochondrial PCGs.
