# Supplementary material for: Mitochondrial Genome of Strophopteryx fasciata (Plecoptera: Taeniopterygidae), with a Phylogenetic Analysis of Nemouroidea
Source: Genes (Basel). 2022 Jun 22;13(7):1116. doi: 10.3390/genes13071116 (PMC9322138; doi:10.3390/genes13071116)
Supplement: Supplementary file 1 [file genes-13-01116-s001.zip › genes-1762865-supplementary.pdf]

Supplementary Materials

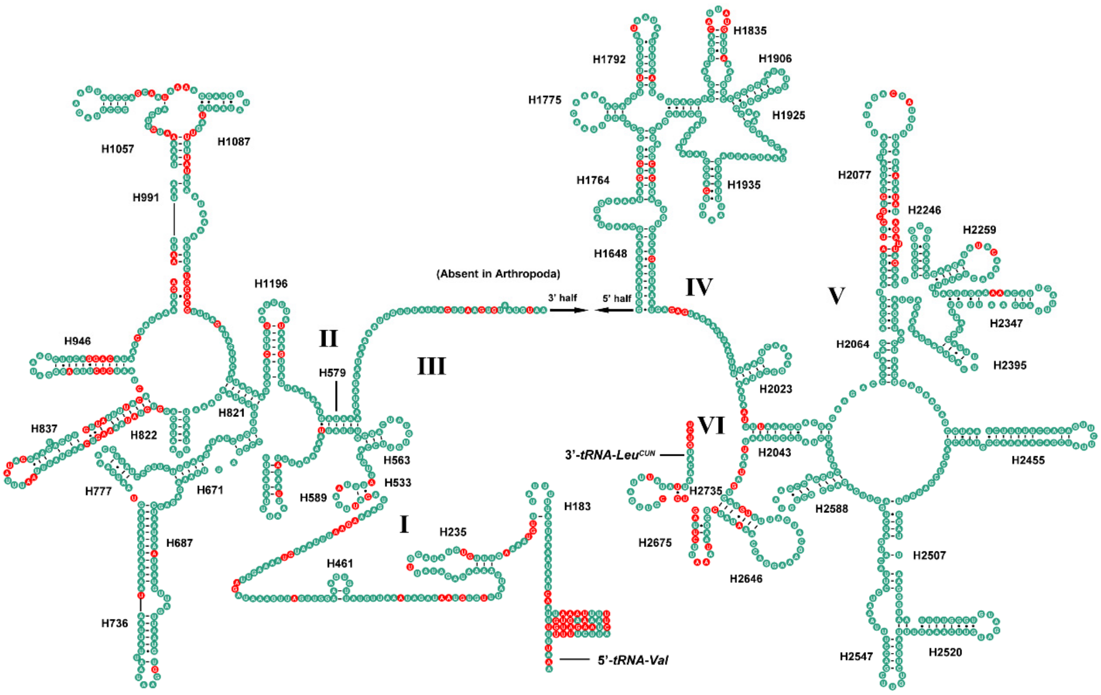

Figure S1. Predicted secondary structure of the *lrRNA* gene in *S. fasciata* mitogenome.

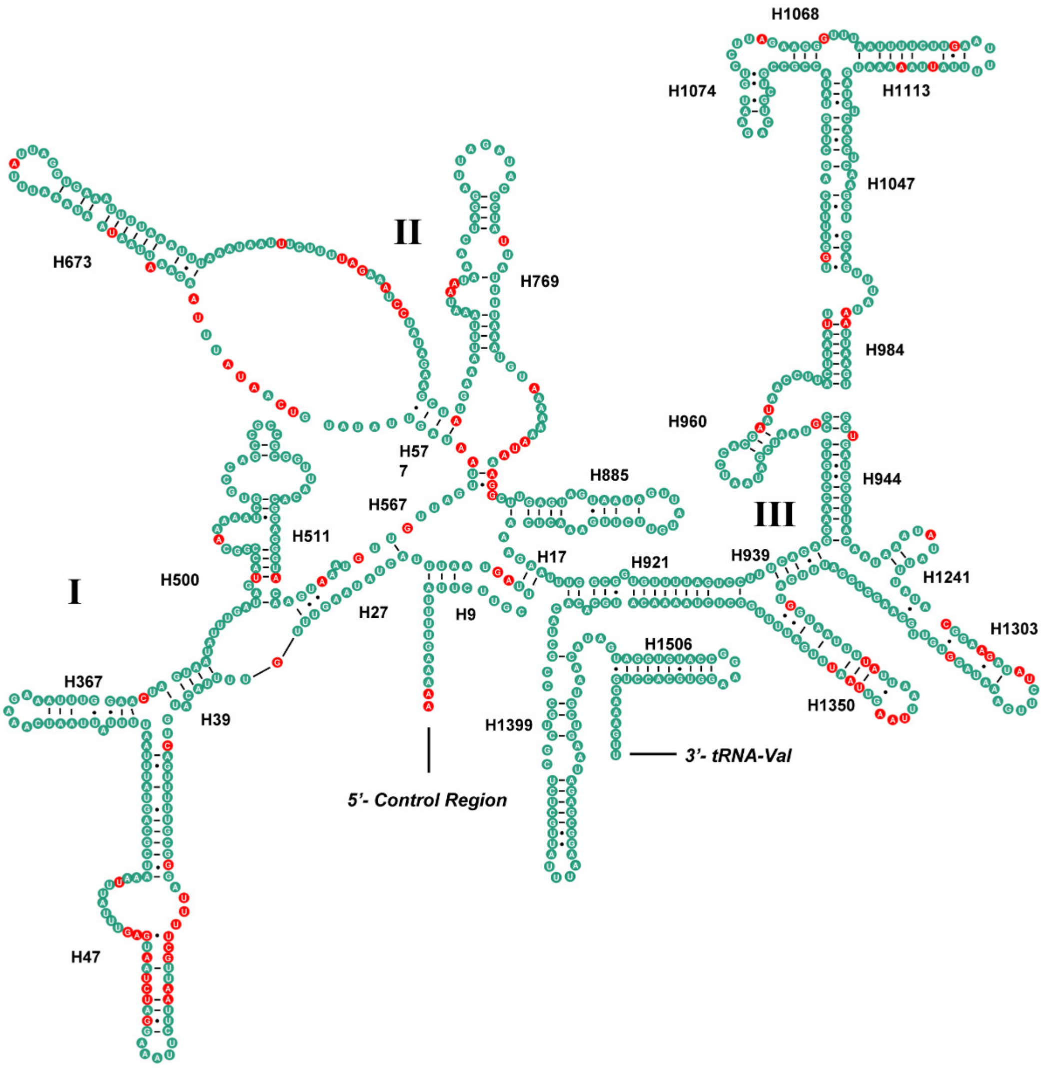

Figure S2. Predicted secondary structure of the *srRNA* gene in *S. fasciata* mitogenome.

Table S1. Organization of the *S. fasciata* mitochondrial genome.

Gene	Direction	Location	Size	Anticodon or start/stop codon	Intergenic nucleotides
*tRNA^Ile^*	F	1-67	67	30-32 GAT	
*tRNA^Gln^*	R	65-133	69	101-103 TTG	-3
*tRNA^Met^*	F	133-203	71	163-165 CAT	-1
ND2	F	204-1,238	1,035	ATG/TAA	0
*tRNA^Trp^*	F	1,237-1,304	68	1,267-1,269 TCA	-2
*tRNA^Cys^*	R	1,297-1,361	65	1,329-1,331 GCA	-8
*tRNA^Tyr^*	R	1,362-1,427	66	1,394-1,396 GTA	0
COI	F	1,420-2,959	1,540	ATT/T-	-8
*tRNA^Leu(UUR)^*	F	2,960-3,025	66	2,989-2,991 TAA	0
COII	F	3,031-3,718	688	ATG/T-	5
*tRNA^Lys^*	F	3,719-3,789	71	3,749-3,751 CTT	0
*tRNA^Asp^*	F	3,789-3,856	68	3,818-3,820 GTC	-1
ATP8	F	3,857-4,015	159	ATT/TAA	0
ATP6	F	4,009-4,686	678	ATG/TAA	-7
COIII	F	4,686-5,474	789	ATG/TAA	-1
*tRNA^Gly^*	F	5,474-5,539	66	5,503-5,505 TCC	-1
ND3	F	5,540-5,893	354	ATT/TAG	0
*tRNA^Ala^*	F	5,892-5,955	64	5,921-5,923 TGC	-2
*tRNA^Arg^*	F	5,956-6,019	64	5,985-5,987 TCG	0
*tRNA^Asn^*	F	6,020-6,087	68	6,050-6,052 GTT	0
*tRNA^Ser(AGN)^*	F	6,088-6,155	68	6,114-6,116 GCT	0
*tRNA^Glu^*	F	6,157-6,221	65	6,186-6,188 TTC	1
*tRNA^Phe^*	R	6,224-6,290	67	6,256-6,258 GAA	2
ND5	R	6,291-8,022	1,732	ATG/T-	0
*tRNA^His^*	R	8,023-8,090	68	8,058-8,060 GTG	0
ND4	R	8,090-9,430	1,341	ATG/TAA	-1
ND4L	R	9,424-9,720	297	ATG/TAA	-7
*tRNA^Thr^*	F	9,723-9,787	65	9753-9755 TGT	2
*tRNA^Pro^*	R	9,788- 9,853	66	9,821-9,823 TGG	0
ND6	F	9,856-10,380	525	ATG/TAA	2
CytB	F	10,380-11,516	1,137	ATG/TAG	-1
*tRNA^Ser(UCN)^*	F	11,515-11,584	70	11,546-11,548 TGA	-2
ND1	R	11,601-12,551	951	TTG/TAG	16
*tRNA^Leu(CUN)^*	R	12,553-12,618	66	12,587-12,589 TAG	1
lrRNA	R	12,619-13,952	1,334		0
*tRNA^Val^*	R	13,953-14,023	71	13,988-13,990 TAC	0
srRNA	R	14,024-14,820	797		0
Control region		14,821-15,527	707		0

Table S2. Codon number and RSCU in the *S. fasciata* mitochondrial PCGs.

Codon	Count	RSCU	Codon	Count	RSCU	Codon	Count	RSCU	Codon	Count	RSCU
UUU(F)	227	1.44	UCU(S)	94	2.24	UAU(Y)	99	1.29	UGU(C)	36	1.64
UUC(F)	89	0.56	UCC(S)	17	0.4	UAC(Y)	55	0.71	UGC(C)	8	0.36
UUA(L)	343	3.18	UCA(S)	90	2.14	UAA(*)	0	0	UGA(W)	94	1.81
UUG(L)	62	0.57	UCG(S)	9	0.21	UAG(*)	0	0	UGG(W)	10	0.19
CUU(L)	117	1.08	CCU(P)	78	2.11	CAU(H)	57	1.5	CGU(R)	19	1.23
CUC(L)	26	0.24	CCC(P)	20	0.54	CAC(H)	19	0.5	CGC(R)	5	0.32
CUA(L)	86	0.8	CCA(P)	38	1.03	CAA(Q)	70	1.73	CGA(R)	26	1.68
CUG(L)	14	0.13	CCG(P)	12	0.32	CAG(Q)	11	0.27	CGG(R)	12	0.77
AUU(I)	241	1.7	ACU(T)	86	1.59	AAU(N)	117	1.5	AGU(S)	65	1.55
AUC(I)	42	0.3	ACC(T)	34	0.63	AAC(N)	39	0.5	AGC(S)	21	0.5
AUA(I)	127	1.36	ACA(T)	82	1.52	AAA(K)	22	0.6	AGA(S)	40	0.95
AUG(M)	60	0.64	ACG(T)	14	0.26	AAG(K)	51	1.4	AGG(S)	0	0
GUU(V)	99	1.72	GCU(A)	119	2.1	GAU(D)	48	1.39	GGU(G)	61	1
GUC(V)	33	0.57	GCC(A)	45	0.79	GAC(D)	21	0.61	GGC(G)	13	0.21
GUA(V)	74	1.29	GCA(A)	54	0.95	GAA(E)	59	1.55	GGA(G)	97	1.58
GUG(V)	24	0.42	GCG(A)	9	0.16	GAG(E)	17	0.45	GGG(G)	74	1.21
